# Atorvastatin Potentially Reduces Mycobacterial Severity through Its Action on Lipoarabinomannan and Drug Permeability in Granulomas

**DOI:** 10.1128/spectrum.03197-22

**Published:** 2023-01-31

**Authors:** Kusuma Sai Davuluri, Amit Kumar Singh, Ajay Vir Singh, Pooja Chaudhary, Sunil Kumar Raman, Shweta Kushwaha, Shoor Vir Singh, Devendra Singh Chauhan

**Affiliations:** a Department of Microbiology and Molecular Biology, ICMR, National JALMA Institute for Leprosy and Other Mycobacterial Diseases, Tajganj, Agra, India; b Division of Pharmaceutics and Pharmacokinetics, CSIR, Central Drug Research Institute, Lucknow, India; c Department of Biotechnology, GLA University, Mathura, India; Institut Pasteur

**Keywords:** chemokines, drug distribution, statins, vasculature, tuberculosis, atorvastatin, drug penetration, *Mycobacterium tuberculosis*, granuloma

## Abstract

The majority of preclinical research has shown that Mycobacterium tuberculosis can modify host lipids in various ways. To boost its intramacrophage survival, M. tuberculosis causes host lipids to build up, resulting in the development of lipid-laden foam cells. M. tuberculosis binds to and enters the macrophage via the cell membrane cholesterol. Aggregation of cholesterol in the cell wall of M. tuberculosis and an increase in vascularity at the granuloma site reduce the permeability of rifampicin and isoniazid concentrations. However, very few studies have assessed the effect of statins on drug penetration. Here, we used atorvastatin, a 3-hydroxy-3-methylglutaryl coenzyme A (HMG-CoA) reductase inhibitor, to observe its effect on the bacterial burden by increasing the drug concentration at the infection site. We looked into how atorvastatin could be used in conjunction with first-line drugs to promote drug permeation. In this study, we detected an accumulation of drugs at the peripheral sites of the lungs and impaired drug distribution to the diseased sites. The efficacy of antituberculosis drugs, with atorvastatin as an adjunct, on the viability of M. tuberculosis cells was demonstrated. A nontoxic statin dosage established phenotypic and normal granuloma vasculature and showed an additive effect with rifampicin and isoniazid. Our data show that statins help to reduce the tuberculosis bacterial burden. Our findings reveal that the bacterial load is connected with impaired drug permeability resulting from lipid accumulation in the bacterial cell wall. Statin therapy combined with antituberculosis medications have the potential to improve treatment in tuberculosis patients.

**IMPORTANCE** Mycobacterium tuberculosis binds to and enters the macrophage via the cell membrane cholesterol. M. tuberculosis limits phagosomal maturation and activation without engaging in phagocytosis. Aggregation of cholesterol in the cell wall of M. tuberculosis and an increase in the vascularity at the granuloma site reduce the permeability of rifampicin and isoniazid concentrations. However, very few studies have assessed the effect of statins on drug penetration, which can be increased through a reduction in cholesterol and vascularity. Herein, we used atorvastatin, a 3-hydroxy-3-methylglutaryl coenzyme A (HMG-CoA) reductase inhibitor, to observe its effect on bacterial burden through increasing the drug concentration at the infection site. Our main research goal is to diminish mycobacterial dissemination and attenuate bacterial growth by increasing drug permeability.

## INTRODUCTION

Tuberculosis (TB) is a contagious infection caused by Mycobacterium tuberculosis that mostly affects the lungs (1). According to World Health Organization (WHO) data released on 21 March 2021, fewer cases of tuberculosis were recorded in 2020 as a result of the new coronavirus disease 2019 (COVID-19) pandemic, which resulted in a half million extra deaths from the disease globally (2, 3). The treatment of tuberculous meningitis caused by rifampicin (RIF)-resistant or multidrug-resistant (MDR) strains is best guided by medication susceptibility data and the ability of TB medicines to reach the infectious site (4, 5). Few data are available on the mechanisms of drug penetration into the infection site. It is advised that drugs used for the regimen have strong central nervous system (CNS) penetration capabilities in individuals with MDR/extensively drug-resistant (XDR) TB meningitis (6). Pyrazinamide and isoniazid (INH) enter the infection site extremely efficiently, with larger dosages achieving sufficient MICs in cerebrospinal fluid.

To reduce the dissemination of infection and to increase drug permeation, invention of an efficient therapy is required. During M. tuberculosis infection, infected host cells undergo phagocytosis, and changes in lipid metabolism are observed as a result of cholesterol upregulation (7). M. tuberculosis bacillus viability is reduced when cholesterol levels are low. Statins act as competitive agonists, binding to the active site of the 3-hydroxy-3-methylglutaryl coenzyme A (HMG-CoA) reductase enzyme and preventing the true substrate (HMG-CoA) from binding (8). M. tuberculosis cells bind to and enter the macrophage via cholesterol in the host cell membrane. Lipoarabinomannan (LAM) surrounding the M. tuberculosis cell arrests phagosome formation, and the cell escapes lysis. Although studies show that statins as an adjuvant can improve the efficacy of first-line TB drugs, relatively little research has been conducted to determine how statins interact with drug penetration mechanisms. Statin-mediated cholesterol inhibition promoted the development of M. tuberculosis phagosomes containing early endosome antigen 1 (EEA1) and lysosome-associated membrane protein (LAMP3+), lowering the mycobacterial loads in macrophages (9). LAM is a lipid component of the mycobacterial cell wall that is crucial for both the innate immune response to infections and pathogenicity. LAM arrests the phagosomal maturation and increases the bacterial burden. The participation of LAM in blocking phagosome processes may be related to its partitioning into host cell endomembranes (10), with its highest activity occurring at the source of origin, the mycobacterial phagosome (8). The ability of M. tuberculosis cells to cause phagosome maturation arrest is dependent on membrane-associated lipids that act as tags, attracting and directing the membrane trafficking machinery (11). Slow antibiotic penetration through the cell wall has been proposed as a contributing factor to the intrinsic drug resistance of M. tuberculosis (12). During M. tuberculosis infection, anti-LAM antibodies are produced. Guinea pigs that were treated with antimicrobial drugs showed much lower antibody titers against LAM and capsular arabinomannan. Apart from LAM, vascularity is also one of the factors that affect drug permeability. Vascular endothelial growth factor (VEGF) has been shown to be a crucial growth factor in the creation of blood vessels (13). Atorvastatin, pravastatin, and cerivastatin have been proven to scavenge oxygen-derived free radicals (14, 15).

As a result, statins may have direct vasculoprotective benefits that are not dependent on cholesterol. Recently, it has been proposed that statins may also influence VEGF production and, as a result, angiogenesis. Indeed, lovastatin reduced VEGF synthesis in transformed fibroblasts at micromolar doses (16). In this research, we proposed that atorvastatin potentiates the antibacterial effects of anti-TB drugs. Treatment with atorvastatin lowers VEGF levels, which are implicated in the maintenance of angiogenesis. In addition to anti-TB medication, blocking neovascularization with anti-VEGF may be a viable option for controlling the spread of M. tuberculosis infection to other organs (17). We demonstrate in this work that the impact of statins on cholesterol caused a difference in the infection load due to reduced LAM and angiogenic support at the granuloma. This is a preliminary study, which shows that atorvastatin treatment lowers aberrant vasculature and promotes medication absorption. Our next study will analyze the number of phagosomes at different periods during the therapy.

## RESULTS

### In guinea pigs, atorvastatin and anti-TB drugs have a synergistic bactericidal action.

**FIG 1:**
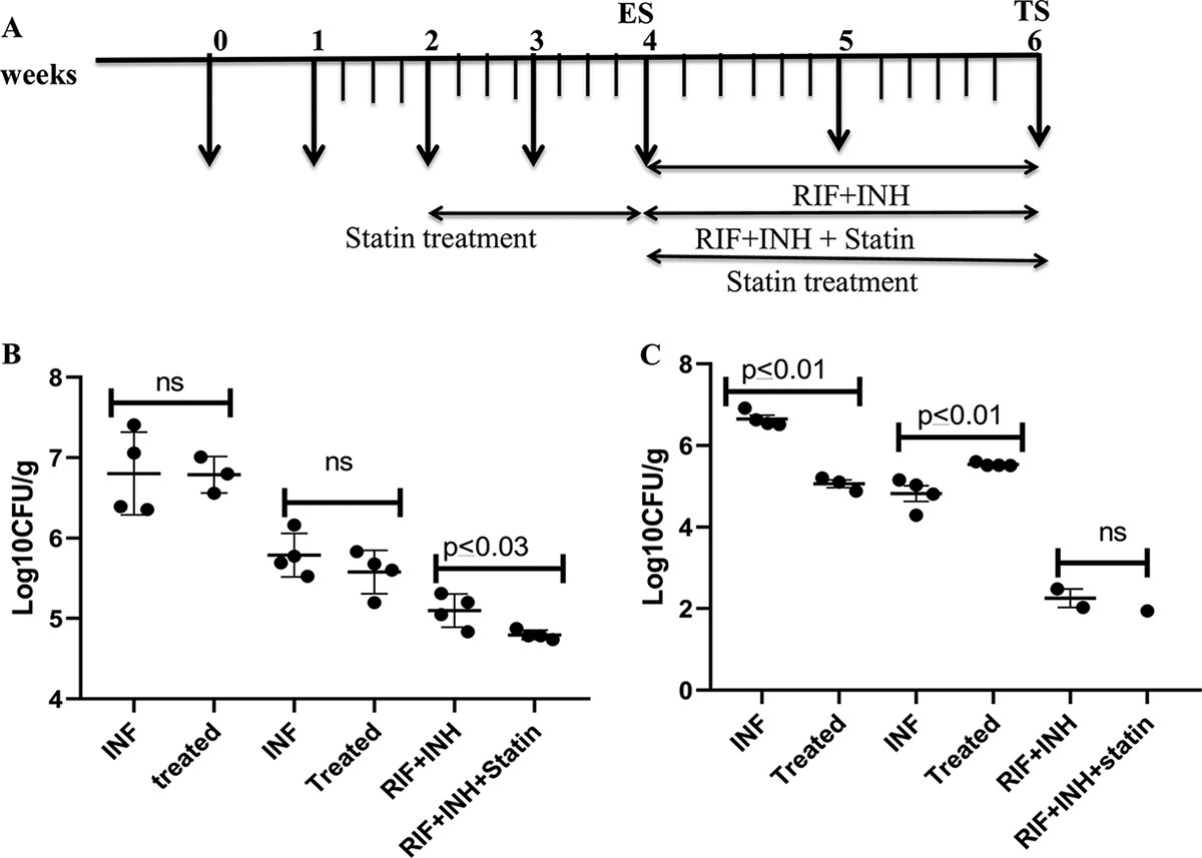
In guinea pigs, atorvastatin improved the anti-TB activity of the first-line regimen. (A) Timeline showing the treatments given. Compared to the control, atorvastatin 5 mg/kg administered alone after 2 weeks of aerosol infection had no effect on the course of acute infection at lungs. When combined with rifampicin/isoniazid and atorvastatin, the lung bacillary load was dramatically reduced compared to conventional therapy alone. Results are shown as the mean CFU plus the SD of lung (B) and spleen (C) per gram (log_10_) from five animals per group, at each time point, or as the proportion of lung exhibiting inflammatory involvement. Dissemination of bacilli was reduced in the spleen in atorvastatin treated guinea pigs, when compared with *M. tuberculosis* infected guinea pigs without treatment, but not significantly. Statistical comparison between the groups was carried out by employing an unpaired *t* test. INF, infection; ns, not significant. Es, early stage; TS, terminal stage.

In the second week after infection with M. tuberculosis H_37_Rv cells in the presence of atorvastatin, guinea pigs showed a considerably decreased intracellular bacterial load in the spleen (Fig. 1A) (*P* ≤ 0.05). Guinea pigs given atorvastatin had substantially lower levels of LAM than in the infected control groups (*P* ≤ 0.05). Since lipid body formation in macrophages has been linked to M. tuberculosis growth restriction and phenotypic drug tolerance, we wanted to see if atorvastatin could make intracellular bacilli more sensitive to rifampicin (RIF) and isoniazid (INH). Next, we investigated the anti-TB activity of a combination regimen combining the first-line drugs rifampicin and isoniazid, as well as atorvastatin, in guinea pigs with chronic TB infection. A total of 2 weeks of atorvastatin treatment was given to guinea pigs, commencing 2 weeks after infection. After 2 weeks of therapy, no difference in CFU was observed (*P* ≥ 0.05), indicating that atorvastatin alone lacks anti-TB action during the acute stage of infection (Fig. 1A). In another study, infection was allowed to spread for 4 weeks before starting therapy with rifampicin/isoniazid or rifampicin plus isoniazid plus atorvastatin (Fig. 1A). After 2 weeks of therapy, the atorvastatin-containing combination regimen had stronger activity against bacilli in the lungs than rifampicin/isoniazid alone, indicating that statins may boost the activity of first-line regimens (Fig. 1B). In the spleen, the dissemination of infection was reduced in guinea pigs treated with atorvastatin as adjunct with RIF plus INH. The lung histology results did not show any difference (Fig. 1).

### Statins that inhibit LAM expression may have a role in drug permeability.

**FIG 2:**
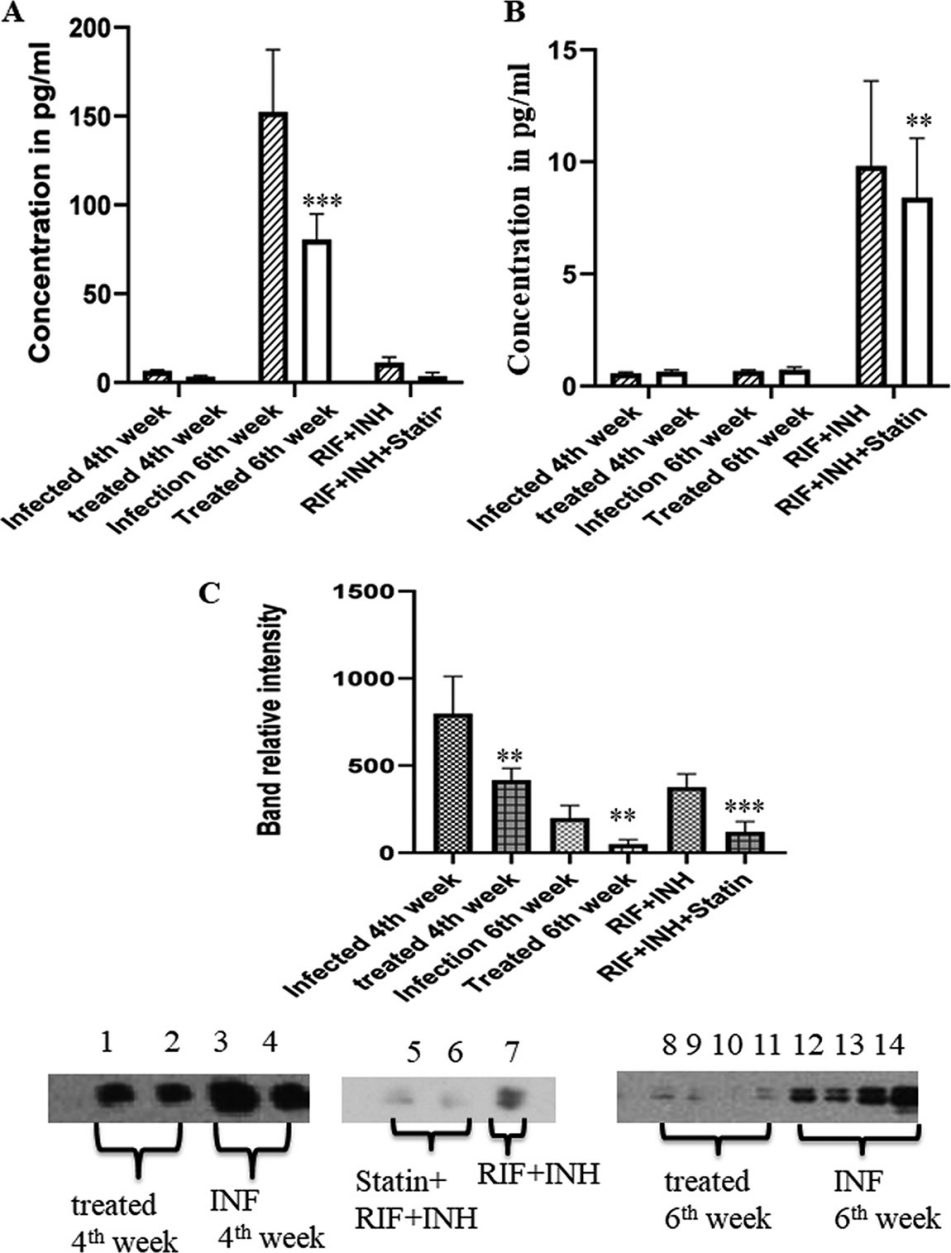
Atorvastatin downregulates LAM expression. (A) Graph showing the antigen levels of LAM in different groups. One-way ANOVA was used to compare the treated conditions to the infection control preceding multiple comparisons. At 2 and 4 weeks, the statin-treated groups showed a significant (*P* ≤ 0.03) reduction in LAM expression. (B) Graph showing the anti-LAM antibody responses in different groups, revealing enhanced colocalization of LAM in M. tuberculosis-infected guinea pigs. M. tuberculosis colocalizes quantitatively with LAM. Following atorvastatin therapy, the markers significantly (*P* ≤ 0.01) decreased. There was a considerable increase in the LAM profile between 4 and 6 weeks after M. tuberculosis infection (*P* ≤ 0.01, based on an unpaired *t* test; *n* = 5). (C) Quantitative analysis of Western blot bands. According to a qualitative analysis, expression of LAM antigens was considerably reduced in the treated groups (lanes 8 to 12) compared to the M. tuberculosis-infected groups.

M. tuberculosis interacts with cholesterol-rich membrane regions during internalization. This is reflected in the buildup of lipid-rich structures within phagosomes in the periphery of bacteria, allowing the bacilli to avoid the host protective process. It was recently established that macrophages derived from simvastatin-treated mice kill M. tuberculosis more effectively via phagosomal maturation and autophagy (18). It is widely established that cellular lipid LAM metabolism changes following M. tuberculosis phagocytosis by host cells. This causes an increase in cholesterol, aliphatic lipid absorption, and *de novo* synthesis (19). Due to the increased LAM concentration, drug penetration might be reduced and might involve phagosomal maturation, as stated by a few previous studies. To observe the effect of atorvastatin on LAM expression, after sacrificing the guinea pigs at a specific time point, the lung tissue cells were rinsed with medium before being lysed in ice-cold radio immunoprecipitation assay (RIPA) buffer containing protease inhibitors. Soluble lysate fractions were recovered, and blots were probed with LAM antibodies. The quantitative expression of LAM was also observed in different groups using an enzyme-linked immunosorbent assay (ELISA). We looked at whether statin induction decreases cellular LAM levels, possibly altering the evasion mechanism induced in Mycobacterium tuberculosis (Fig. 2).

### Statin treatment reduced VEGF expression.

**FIG 3:**
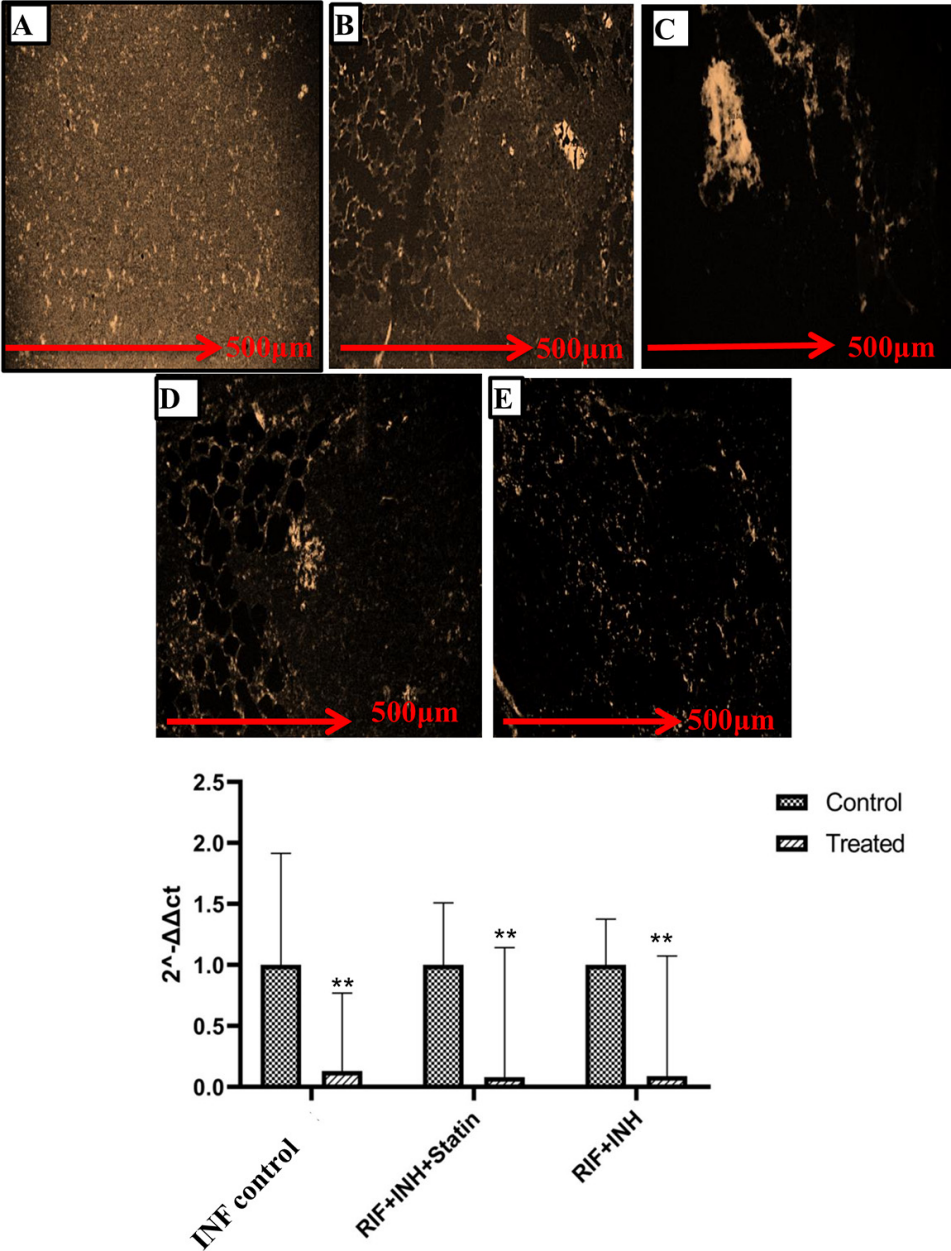
Immunofluorescence-stained slides showing the expression of VEGF in different groups. One-way ANOVA was used to compare the treated conditions to the infection control preceding multiple comparisons. (A) Vascularity in the lungs is normal in the healthy guinea pigs. (B) VEGF expression was significantly upregulated in the M. tuberculosis-infected guinea pig lungs. (C) Treatment with atorvastatin reduced the expression of VEGF significantly (*P* < 0.01). (D, E) Treatment with anti-TB drugs attenuated the level of VEGF antibodies (VEGFA) (D), which was further reduced in guinea pigs treated with anti-TB drugs and atorvastatin combined (E). (F) Similar results were observed for VEGF mRNA expression. VEGF expression was dramatically decreased after treatment with atorvastatin (*P* ≤ 0.01). VEGF mRNA levels were further reduced in guinea pigs treated with anti-TB drugs and the atorvastatin combination, bringing the angiogenic factor levels back to normal. ANOVA was performed for multiple comparisons, and significant (*P* ≤ 0.05) changes were found. *C_T_*, threshold cycle.

Anti-VEGF antibodies can increase the distribution of a low-molecular-weight tracer and establish more architecturally and functionally normal granuloma vasculature in cancer and eye illnesses. Existing infections may be treated therapeutically with the VEGFR antagonist pazopanib, which reduces vascularization and bacterial burdens, and granuloma vascularization reduces bacterial dispersion from established infections. Expression of VEGF was found in the homogenized lung tissues of the guinea pigs. In the afflicted areas of the lung tissue, VEGF expression was extremely high. Guinea pigs were infected with M. tuberculosis and given atorvastatin treatment for 2 weeks. The guinea pigs were then sacrificed, and the VEGF expression in lung sections was examined (Fig. 3).

### Atorvastatin treatment improves the distribution of rifampicin and isoniazid.

**FIG 4:**
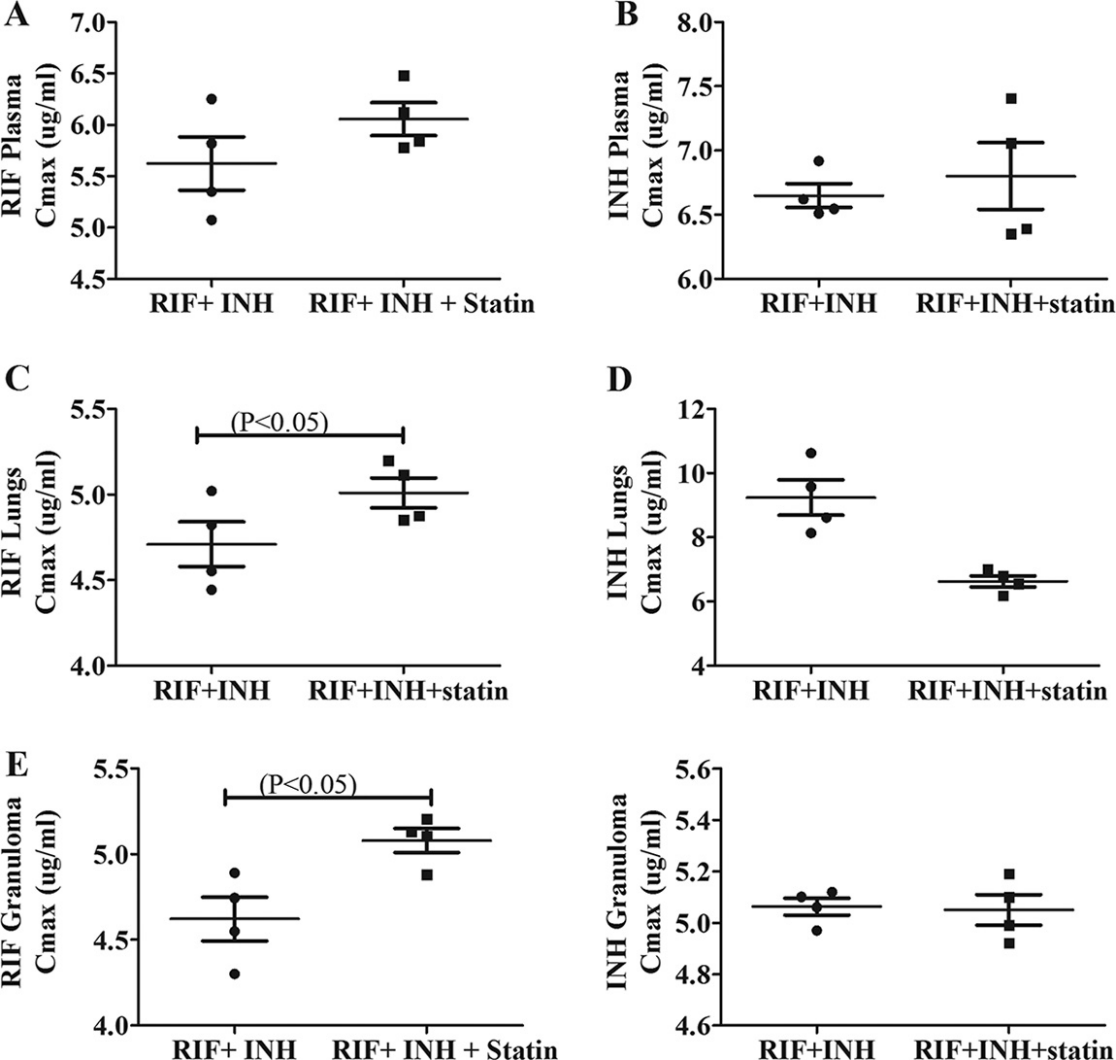
To reach the bacilli inside the macrophages, antituberculosis drugs must overcome barriers such as lesions in the lungs, with higher cellular composition and vascularization. From the blood, the drug enters the interstitial space of lesions and then penetrates them. (A to E) The distribution of drug concentration decreased in plasma (A, B), lungs (C, D), and granulomas (E), respectively; atorvastatin-treated guinea pigs combined with first-line drugs showed effective penetration of the drugs into the lesions. *P* ≤ 0.01 was considered statistically significant. In plasma and lung tissue, no significant difference in isoniazid concentration was seen. *C*_max_, maximum concentration of drug in serum.

Plasma RIF and INH were estimated simultaneously after extraction. Analysis was performed using a C8 column at 267 nm. The retention times of rifampicin and isoniazid were 3 and 4.5 min. Atorvastatin treatment improves drug delivery in granulomas. M. tuberculosis lesions on the lungs were isolated from the right apex region, and caudal lung tissue was collected for the drug estimation. Normalization of the vasculature and refining the vascular perfusion can increase the distribution of first-line drugs in granulomas. Atorvastatin treatment normalized the granuloma vasculature, and we investigated if the drug delivery could be improved by reducing the thickness of the vascularity. After 2 weeks of atorvastatin treatment, the rifampicin and isoniazid delivery to granulomatous lesions was significantly higher than in the controls (*P* ≤ 0.05) (Fig. 4).

### Increased levels of alanine aminotransferase in atorvastatin-treated guinea pigs.

**FIG 5:**
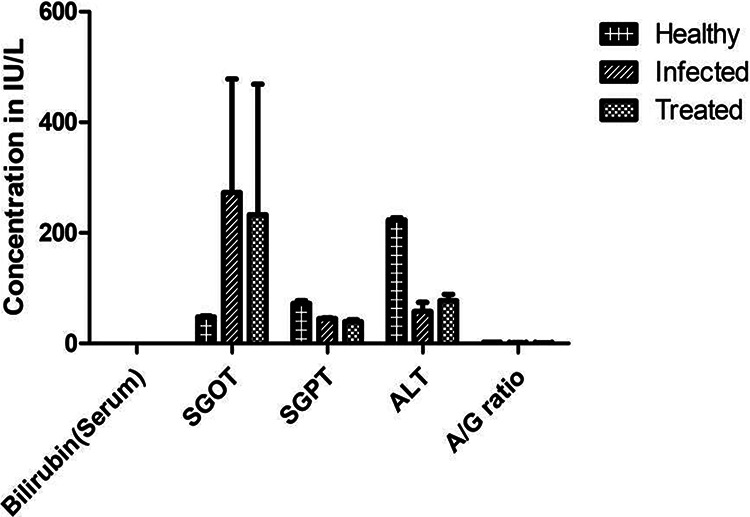
Drug action is always involved in the biochemical processes taking place in the body, so the biochemical parameters of liver function tests (LFTs) were measured after atorvastatin therapy, and the risk factors were observed. Biochemical testing of serum samples from atorvastatin-treated M. tuberculosis-infected guinea pigs provided critical insights into the drug. Biochemical variables were compared between three groups of guinea pigs. We evaluated plasma for liver enzymes to determine the status of liver functions during drug therapy. Alanine aminotransferase (ALT) levels in atorvastatin-treated guinea pigs were significantly increased compared to M. tuberculosis-infected guinea pigs without treatment. SGOT, serum glutamic-oxaloacetate transaminase; SGPT, serum glutamate pyruvate transaminase; A/G ratio, albumin/globulin ratio.

The biochemical parameters of liver function tests (LFTs) were measured after 2 weeks of atorvastatin treatment, and the risk factors were recorded. Confirming the previous results, atorvastatin increased the alanine aminotransferase (ALT) levels, which decreased after the discontinuation of the drug. After 2 weeks of treatment, atorvastatin induced the acute elevation of only one hepatic enzyme. The other LFT baseline enzymes were normal in all groups. Aspartate aminotransferase was considerably elevated during the atorvastatin treatment (Fig. 5).

## DISCUSSION

M. tuberculosis induces the formation of lipid droplets in host cells, demonstrating that host-derived cholesterol is the most abundant lipid component within these organelles (20). *In vitro* studies have already shown that host cholesterol accumulation in the M. tuberculosis cell wall is related to a decrease in rifampicin permeability, which might explain the additive effect observed between statins and anti-TB drugs (21). Penetration of antibiotics depends upon several mechanisms, such as lower cellular permeability, efflux pumps, drug-modifying enzymes, and drug-neutralizing protein (22). In an attempt to analyze the causes of underlying resistance patterns, it has been proposed that the permeability of antituberculosis medications is dependent across mycobacterial species. Antibiotic resistance may be linked to mycolic acids, a possible permeability barrier (23). This is the reason why researchers now focus on cell-penetrating peptides (24, 25). It is very important to address the issue of what the cell wall of drug-resistant M. tuberculosis strains looks like (26). Despite the fact that M. tuberculosis shares 40% of human enzymes, statins do not inhibit mycobacterial HMG-CoA reductase. More importantly, the higher atorvastatin concentrations used in this investigation (5 mg/kg) did not show a significant reduction in guinea pigs. Parihar and colleagues have established that statins can inhibit M. tuberculosis infection and they postulated that this effect is due to phagolysosomal halt in M. tuberculosis. In our study, atorvastatin demonstrated comparable mycobactericidal action against M. tuberculosis. Our *in vivo* findings corroborate *in vitro* findings in the literature with substantial differences from the control groups identified only in mice receiving a statin dosage (5 mg/kg) (27). Our data demonstrate that statins can inactivate M. tuberculosis *in vitro* and boost the antibacterial action of the anti-TB drugs RIF and INH. According to a quantitative analysis, the atorvastatin-treated guinea pigs demonstrated significantly reduced LAM. Reduced Western blot and densitometric analysis of the bands indicated the protein levels. These findings imply that statins modulate the host immunological responses by downregulating the LAM levels. Surprisingly, statin medication prior to M. tuberculosis infection reduced LAM and might boost autophagy in macrophages. Statins, which have more than 100 million prescriptions globally, decrease cholesterol in those with cardiovascular disease (28). Statins block HMG-CoA reductase, the mevalonate pathway’s rate-controlling enzyme (8). Furthermore, statins have wide immune-modulatory and anti-inflammatory characteristics, making them potentially useful as host-directed therapies (HDTs) against infectious illnesses (29). Statins decreased the M. tuberculosis load in mice by promoting autophagy and phagosome maturation and lowering the pulmonary pathology, with fewer and smaller lesions, indicating a potential for statins as an HDT in TB (30). Others have observed that statins as a supplemental therapy shortened the time to TB cure and reduced the mouse lung pathology, resulting in smaller lung lesions (31). Adjunctive statins reduced the lung CFU and the proportion of lung surface area implicated in inflammation in C3HeB/FeJ (Kramnik) mice (32). Several population-based studies found that statin use was related to a lower incidence of tuberculosis (33, 34). Pravastatin is now being studied in a clinical phase 2b dose-finding trial in people with tuberculosis. Peak concentrations of 3 to 8 mg/L appear in blood between 1 and 2 h after ingestion of 300 mg of isoniazid; it diffuses into all body tissues, including cerebrospinal fluid (35). Rifampicin has bactericidal activity against dividing mycobacteria and also has some activity against nondividing bacilli. This drug is readily absorbed within 2 to 4 h of ingestion of a dose of 600 mg; peak plasma concentrations may reach 7 to 10 mg/L (36). Reduced vasculature is widely recognized as one of the main reasons for the resistance of solid cancer tumors and granuloma to drug treatment (37). Decreasing drug levels are observed as the distance from blood vessels increases (38). Interestingly, reduced vasculature also impairs the supply of oxygen and nutrients, which results in a decrease in cell proliferation and an increase in drug tolerance (39). The vascular region is destroyed in the caseous center of necrotic lesions and cavities, leading not only to a reduced drug supply but also to the metabolic quiescence of bacterial cells, as a consequence of reduced oxygen and nutrient supplies, and failed immunity owing to poor access by circulating T lymphocytes (40). At the center of necrotic granulomas, quiescent extracellular bacilli are found in large numbers; drugs must diffuse from the vasculature and group of immune cells that border the necrotic center and disperse throughout the entire caseous region without any transport mechanisms. The lack of drug distribution to compartments where large numbers of nonproliferating bacteria reside highlights the importance of assessing drug penetration at these sites of M. tuberculosis infection. At the infectious site, the diversified vascular supply within the granuloma hinders drug delivery (41, 42). Both incipient and established infections could be treated, with reductions in vascularization and bacterial burdens (43). This treatment enhanced 2-fold increases in the therapeutic efficacy of a drug, with no evidence of increased toxicity (44). Targeting the drug penetration mechanisms could be a novel therapeutic strategy for increasing the index of chemotherapeutic drugs. Our data demonstrate that disseminated tuberculosis is associated with elevated levels of LAM and angiogenic factors. Previous studies detected LAM as a biomarker in disseminated tuberculosis (45). Anti-VEGF therapy using statins has the potential to enhance treatment in patients with TB (46–48). In this study, we revealed, for the first time to our knowledge, in a guinea pig model of TB, that treatment with atorvastatin normalizes the vasculature and enhances the delivery of first-line drugs of TB during the normalization of vascularity at granuloma. Our findings could be rapidly tested to enhance TB treatment, shorten the duration of treatment, and control the dissemination or recurrence of infection. The main limitation of the study is its smaller sample size; in addition, we were unable to elucidate the exact mechanism of drug penetration. This preliminary study demonstrated an efficient short-term therapy for tuberculosis.

## MATERIALS AND METHODS

### Ethics statement.

All animal studies were performed in accordance with ARRIVE guidelines and instructions. Experiments involving the use of guinea pigs were approved by the Institutional Animal Ethics Committee of the National JALMA Institute for Leprosy and Other Mycobacterial Diseases (NJIL&OMD; Agra, India). Lala Lajpat Rai University of Veterinary and Animal Sciences (Hisar, India) provided healthy outbred Hartley strain male guinea pigs (weight, ~350 g). All experimental animal methods described in the present study were conducted according to the relevant guidelines and regulations for handling laboratory animals approved by CPCSEA, and the research was approved by the institutional Animal Ethics Committee of NJIL&OMD (NJIL&OMD-3-IAEC/2019-08).

### Guinea pigs are infected with M. tuberculosis in an experimental setting.

M. tuberculosis strain H_37_Rv cells, obtained from the repository at the ICMR National JALMA Institute for Leprosy and Other Mycobacterial Diseases (Agra, India), were grown at 37°C in Middlebrook 7H9 medium supplemented with 10% albumin dextrose catalase (ADC) (Difco Laboratories, USA). When it was determined that the culture had reached mid-log phase, it was frozen in aliquots at −70°C until needed. The cultures were diluted in sterile normal saline prior to use, and the bacterial concentration was adjusted to 1 × 10^6^ CFU/mL (49).

The guinea pigs were maintained under specific pathogen-free conditions and provided water and standard feed *ad libitum*. The guinea pigs were aerosol infected with M. tuberculosis strain H_37_Rv using a full-body inhalation exposure system (Glas-Col, USA), calibrated to deliver ~150 CFU to the lung (50). Three animals were sacrificed 1 day after infection, and four guinea pigs were sacrificed 1 month after infection in order to determine the number of bacilli implanted in each lung on the day after infection and the lung bacillary load at the start of treatment, respectively. The number of animals per group was calculated as mentioned in reference 51. Following infection, the animals (*n* = 5/group) were randomly assigned to different groups, and treatment was initiated as described in Table 1. The animals were monitored daily for changes in body weight (weight loss of >10%), reduced food consumption, and subdued behavior. No adverse events requiring euthanasia/death of the animals were noted during the experiments. As the experiment was completed within 6 weeks, no untreated M. tuberculosis-infected animals reached a stage of overtly symptomatic disease. All animal welfare factors were taken into account, including measures to reduce pain and discomfort through the use of anesthesia and particular housing conditions. The guinea pigs were anesthetized with 4 to 5% isoflurane for induction and 1 to 2% for maintenance. After a >60-s exposure to anesthesia, the guinea pigs were monitored until the lack of respiration and high dose and long exposure to anesthesia caused death. Blood was collected under anesthesia from the guinea pigs 4 and 6 weeks after the dose.

**TABLE 1:** Basic experimental scheme^*a*^

Group	Treatment^*b*^	Treatment initiation (weeks postinfection)	Treatment duration (weeks)	Estimation of CFU (weeks postinfection)
INF				4 and 6
INF/statin at 4 weeks	Atorvastatin	2	2	4^*c*^
INF/statin at 6 weeks	Atorvastatin	4	2	6^*c*^
INF/INH+RIF	INH+RIF	4	2	6^*c*^
	Chemotherapy	4		
INF/INH+RIF/statin	INH+RIF plus atorvastatin	4	2	6^*c*^
	Chemotherapy	4		

a INF, infection (day 0) with 150 to 200 CFU of M. tuberculosis by aerosol inhalation.

b Doses: 5 mg/kg atorvastatin, 30 mg kg^−1^ isoniazid (INH), and 50 mg kg^−1^ rifampicin (RIF) daily by oral gavage (52).

c Following a washout period of 3 days.

After a drug washout period of 3 days, blood was collected by cardiac puncture before the final sacrifice under the influence of anesthesia. The guinea pigs were euthanized using a chamber filled with isoflurane vapor until respiration ceased; cessation of the respiratory and cardiovascular movements was verified by observation in room air for at least 10 min before opening the body cavity under aseptic conditions. At each time point, the lungs and spleen of each animal sacrificed were aseptically removed, examined, and photographed for gross pathology. The lungs and spleen were homogenized and plated onto Middlebrook 7H11 plates for CFU enumeration. Although 7H11 is the selective medium for M. tuberculosis, Ziehl-Neelsen (ZN) staining of the organ homogenate and tissue sections was performed for additional confirmation of the identification of the colony as M. tuberculosis. Further, the left caudal lung from the animals was fixed in 10% buffered formaldehyde for histopathology studies.

### Bacterial colony count determination.

The bacterial burden in infected guinea pig lungs and spleens was measured 4 and 6 weeks after infection. Phosphate-buffered saline (PBS) was used to weigh and homogenize the organs. Tenfold serial dilutions of the organ homogenates were plated onto Middlebrook 7H10 (BD & Co.) agar plates containing 10% oleic acid-albumin-dextrose-catalase (OADC) and incubated at 37°C for 21 days. The colonies on the plates were enumerated for determination of the bacterial burden.

### Biochemical parameter testing.

The drug action is always involved in biochemical processes in the body, so the biochemical parameters of liver function tests (LFTs) were measured after the atorvastatin therapy using a fully automatic clinical chemistry analyzer (TBA-120FR Pearl; Abbot). The risk factors were observed during the treatment. Biochemical testing of serum samples from the atorvastatin-treated M. tuberculosis-infected guinea pigs provided critical insights into the drug. The biochemical variables of healthy guinea pigs, M. tuberculosis-infected guinea pigs, and M. tuberculosis-infected atorvastatin-treated guinea pigs were compared.

### Antimycobacterial IgG responses in disseminated tuberculosis.

The levels of anti-LAM antibodies in the plasma of guinea pigs with disseminated tuberculosis (late stage of infection) were determined manually. Microtiter plates were coated with 100 μL of LAM antigen (10 μg/mL) in coating buffer and incubated overnight at 4°C. We selected anti-human antibodies, which have species reactivity for guinea pigs (per the manufacturer). Due to the similarities between humans and guinea pigs, anti-human antibodies are used for guinea pigs (52, 53). The plates were then cleaned three times and allowed to dry at room temperature for 3 h. Each well received a total of 50 μL of serum. After the plates were incubated at 25°C for 2 h, a diluted (1:10,000) anti-human IgG-alkaline phosphatase antibody produced in goat was added. The incubation process was then restarted for 1 h. As a substrate, an OPD (o-phenylenediamine dihydrochloride) buffer was utilized, and the color development was evaluated spectrophotometrically at 405 nm.

### Analysis of LAM antigen expression.

The concentrations of LAM antigen in the lung tissues of all groups of guinea pigs were determined manually by sandwich ELISA. The concentration of LAM antigen expression levels in lung tissue homogenate samples of M. tuberculosis-infected guinea pigs was determined in duplicate using a specific sandwich ELISA. Similarly, we examined this in the statin-treated guinea pigs and observed the LAM expression levels. The concentration of the minimum detection limit for LAM was 15.6 pg/mL. The results were validated following the standard protocol for Western blotting, as described previously (53). LAM expression was normalized using the reference loading control β-actin.

### Drug estimation.

Concentrations of rifampicin and isoniazid in plasma were determined concurrently following an approved protocol (54). The analysis was carried out on a C8 column at 267 nm. Water, methanol containing perchloric acid, and tetrabutyl N-ammonium hydroxide comprised the mobile phase. The drug estimation procedure included deproteinizing the samples with perchloric acid and analyzing the supernatant using a reversed-phase CN column (150 mm) and a UV detector set at 267 nm. The mobile phase was made up of Milli-Q water and methanol with 0.05% perchloric acid and 0.1% tetrabutyl N-ammonium hydroxide. According to the procedure, stock solutions of rifampicin and isoniazid at 1 mg/mL were produced in methanol with ascorbic acid at 1 mg/mL to avoid oxidation. Phosphate buffer, acetonitrile, and isopropyl alcohol were used to make the mobile phase.

### Detection of VEGF antibody expression through immunohistochemistry and qRT-PCR.

Fluorescein isothiocyanate (FITC)-conjugated VEGF antibodies (VEGFA) (American Assay Laboratories, USA) were used for immunofluorescent staining, with DAPI (4′,6-diamidino-2-phenylindole) as the counterstain. Briefly, tissue sections were incubated with anti-VEGF antibodies for 1 h. Excess antibody was removed by washing the sections with phosphate-buffered saline (PBS) at room temperature for 30 min and incubating them with rabbit anti-mouse secondary detection antibody conjugated with horseradish peroxidase (HRP). End product visualization was achieved using DAPI (Sigma, USA). mRNA was extracted from the plasma samples using TRIzol-C (SRL Diagnostics, India) per the manufacturer’s protocol. Reverse transcription-PCR (RT-PCR) was performed using the SYBR green detection kit (Roche Diagnostics, Switzerland) with the Bio-Rad CFX96 real-time thermal cycler for signal detection. Primers were designed using Primer3Plus software, and the sequences are as follows: 5′-GGCCCATCGAGATGCTAGTG-3′ (forward) and 5′-GCCCACAGGGATTTTCTTGC-3′ (reverse). Each reaction was performed in triplicate for each animal with 0.2 mmol/L of each primer and 50 ng of cDNA template. The cycling conditions were as follows. Reverse transcription took 15 min at 50°C, followed by 3 min of initial denaturation at 95°C, 40 cycles of denaturation at 95°C for 10 s, and 30 s of annealing/extension at 60°C. Gene expression was normalized using the RNase P gene as the reference.

### Pharmacokinetic variable calculation and statistical analysis.

GraphPad Prism and Origin software were used for statistical analysis. ImageJ/Fiji software was used to measure VEGFA expression. The differences in CFU counts, LAM expression, and reverse transcription-quantitative PCR (qRT-PCR) results across the groups were analyzed using GraphPad Prism and statistically assessed using one-way analysis of variance (ANOVA) to compare the means of multiple groups and unpaired *t* test for comparison between two groups. A *P* value of ≤0.05 was deemed significant. We interpreted the normal distribution (Gaussian) using the Shapiro-Wilk test, Kolmogorov-Smirnov test, D’Agostino-Pearson test, and Anderson-Darling test using Graph Pad Prism v8. The high-performance liquid chromatography (HPLC) data passed the normality test with a Shapiro-Wilk test alpha value of 0.05, as did the CFU data. The same test was performed for the remaining results, which yielded an alpha value of 0.05 using the Shapiro-Wilk test but showed *n* as too small.

### Data availability.

The data sets used and analyzed in the current research are available on reasonable request from the corresponding author.
